# Development and Characterization of a Bioinspired Bone Matrix with Aligned Nanocrystalline Hydroxyapatite on Collagen Nanofibers

**DOI:** 10.3390/ma9030198

**Published:** 2016-03-15

**Authors:** Hsi-Chin Wu, Tzu-Wei Wang, Jui-Sheng Sun, Yi-Hsuan Lee, Meng-Han Shen, Zong-Ruei Tsai, Chih-Yu Chen, Horng-Chaung Hsu

**Affiliations:** 1 Department of Materials Engineering, Tatung University, Taipei 10461, Taiwan; rita77117@gmail.com (Y.-H.L.); tony800625@gmail.com (M.-H.S.); ray760930@gmail.com (Z.-R.T.); 2 Department of Materials Science and Engineering, National Tsing Hua University, Hsinchu 30013, Taiwan; twwang@mx.nthu.edu.tw; 3 Department of Orthopedic Surgery, National Taiwan University Hospital, Taipei 10041, Taiwan; 4 Department of Biomedical Engineering, National Yang-Ming University, Taipei 11221, Taiwan; aleckc2424@gmail.com; 5 Department of Orthopedics, China Medical University Hospital, Taichung 40447, Taiwan; d4749@mail.cmuh.org.tw

**Keywords:** nanocomposite, hydroxyapatite nanocrystal, biomimetic scaffold

## Abstract

Various kinds of three-dimensional (3D) scaffolds have been designed to mimic the biological spontaneous bone formation characteristics by providing a suitable microenvironment for osteogenesis. In view of this, a natural bone-liked composite scaffold, which was combined with inorganic (hydroxyapatite, Hap) and organic (type I collagen, Col) phases, has been developed through a self-assembly process. This 3D porous scaffold consisting of a c-axis of Hap nanocrystals (nHap) aligning along Col fibrils arrangement is similar to natural bone architecture. A significant increase in mechanical strength and elastic modulus of nHap/Col scaffold is achieved through biomimetic mineralization process when compared with simple mixture of collagen and hydroxyapatite method. It is suggested that the self-organization of Hap and Col produced *in vivo* could also be achieved *in vitro*. The oriented nHap/Col composite not only possesses bone-like microstructure and adequate mechanical properties but also enhances the regeneration and reorganization abilities of bone tissue. These results demonstrated that biomimetic nHap/Col can be successfully reconstructed as a bone graft substitute in bone tissue engineering.

## 1. Introduction

Bone defects and nonunions often occur in clinical orthopedics, while their treatments are more intractable than fractures. Although small bone fractures are capable of self-healing after trauma, massive bone defects or diseased tissues (*i.e.*, osteoporosis, comminuted fracture, osteocarcinoma) still fail to heal properly. Therefore, regeneration of damaged or diseased skeletal tissues with large defects remains a significant challenge in clinical settings. Traditional autografts and allografts are still regarded as the “gold standard” treatment which could accelerate the bone regeneration process. However, the limited quantity of graft sources and the potential risk of infection or loss of function are still major concerns, resulting in restrictions for the treatment strategy. Because of the increasing number of bone graft requirements every year and the limitations of autologous or allologous bone grafts, many attempts have been undertaken to develop synthetic bone replacement materials [1].

Ceramic scaffolds are tough and brittle with interconnected porosity and cell-matrix interactions [2,3]. Alternatively, polymer-based scaffolds can be easily fabricated with different structures, except that they often do not have the desired modulus of elasticity and ultimate strength [4,5,6]. Each unitary composition scaffold has its own advantages and issues. Therefore, a combination of both types of materials to form 3-dimensional porous composite scaffolds has been used in up-to–date research which can enhance the mechanical and biochemical properties of scaffolds for cell migration, proliferation and growth to induce tissue formation in bone tissue engineering [7,8].

Successfully engineered bone grafts must be biocompatible and fit particular minimal mechanical requirements to achieve physiological functionalization *in vivo*. It is necessary to mimic not only the composition of the natural bone but also its ultrastructure to achieve properties that are similar to native bone tissue. Natural bone is composed of organic collagen fiber matrix and inorganic hydroxyapatite nanocrystals mineralized with unique hierarchically organized structures. The mineralized collagen fibrils where plate-like hydroxyapatite crystals are preferentially oriented with their c-axes parallel to the longitudinal axis of the fibrils [9]. Excluding crosslinking, these architecture may be the predominantly reason to achieve the adapted mechanical functions of engineered bone graft [10]. To date, the demand for ideal bone substitute is an unmet need including insufficient mechanical strength, reproducibility and unique hierarchical geometrical arrangement.

Since hydroxyapatite (Hap) and collagen (Col) are the major constituents of human bone, these two components are widely studied as promising materials for bone regeneration [11,12]. Several approaches have been used to prepare a hybrid Hap/Col scaffold for mimicking native bone. One of the common methods is solid-liquid biphase reaction either by addition of synthesized Hap crystal suspension into collagen slurry; alternatively, collagen scaffold was immersed in physiological solutions or media (*i.e.*, simulated body fluid, Earle’s balanced salt solution, Dulbecco’s modified Eagle’s medium) for the *in situ* synthesis of Hap. Although such a Hap/Col composite could improve the stiffness, no nanostructure similar to natural bone was observed [13,14,15]. It is accepted that the mechanical properties of bone are significantly determined by its hierarchical structure. Another approach is liquid unity phase reaction that calcium/ phosphate precursor solution was added into collagen containing solution to precipitate Hap onto the collagen fibril. This biomimetic approach showed intimated interaction of the inorganic and organic components [16,17]. The physiochemical properties of Hap/Col composite are highly affected by fibrillogenesis and mineralization. It is not only influenced by the structural organization of the matrix itself but also by the chemical interactions between the ion precursor and Col matrix under manufacturing process (*i.e.*, temperature, pH, concentration of inorganic ions present in media). The purpose of this study is to develop a one-pot biomineralized synthesis method which facilitates and reproducibly forms engineered bone matrix that fits in with the mechanical needs and nanostructure features of bone, and is suitable for bone regeneration.

## 2. Results

### 2.1. Characterizations of Biomineralized Hydroxyapatite on Collagen

**Figure 1:**
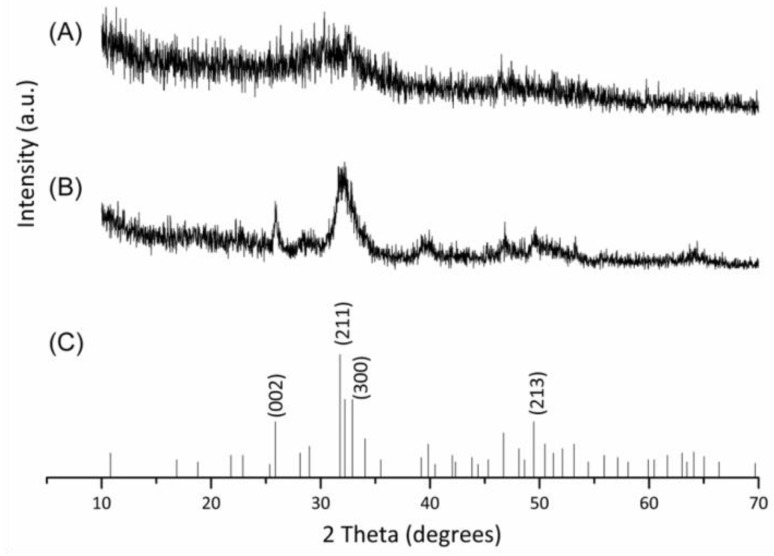
XRD patterns of Hap nanocrystals/ type I collagen composite (nHap/Col composite) scaffold after (**A**) 0.5; (**B**) 24 h reaction; and (**C**) standard reference of Hap (JCPDS No. 09-0432).

Depending on temperature, purities, fabrication and formulation parameters, calcium phosphates can exist in various compound categories [18]. Among of them, Hap is one of the most commonly used phases because of its osteogenic properties and the ability to enhance bone tissues formation [19,20]. In this study, the mineralized collagen composite was successfully synthesized during the biomimetic precipitation process for further analyses. After 0.5 h biomimetic precipitation process, a broadened diffraction peak gradually appeared and possessed a amorphous phase tendency. With the increase of incubation time to 24 h, the amorphous mineral phase was slowly and gradually transformed to crystalline phase. The XRD result showed a similar pattern to the JCPDS reference Hap (Figure 1). Those diffraction peaks were particular corresponding to crystallographic planes (002), (211), (300), and (213) of Hap, respectively. A Hap characteristic pattern was obviously observed that could be attributed to crystallinity reinforcement of Hap. The result reveals that Hap can be successfully crystallized with mineral formation on collagen fibrils during the synthesis process.

Hap and Col are elementary building blocks of bone, demonstrating with a hierarchical structure. The tiny Hap crystals (about 2–4 nm) are plate-shaped, aligned with the collagen matrix, and mostly located in the gap regions of the collagen fibrils [21]. However, several factors (pH, temperature, electrolytes and ionic strength, *etc.*) affect the biomimetic mineralization [22]. Self-assembly of collagen fibrils reconstruction depends on surface charges and isoelectric point (pI) that are influenced by ion species. The presence of divalent ion such as Ca^2+^ will shift pI value to higher pH even if collagen fibrils have varied isoionic points [23]. Therefore, we try to mimic physiological system in reactive condition by starting from an aqueous suspension of calcium precursor incorporating with collagen and phosphate precursor in pH 9 at 37 °C.

**Figure 2:**
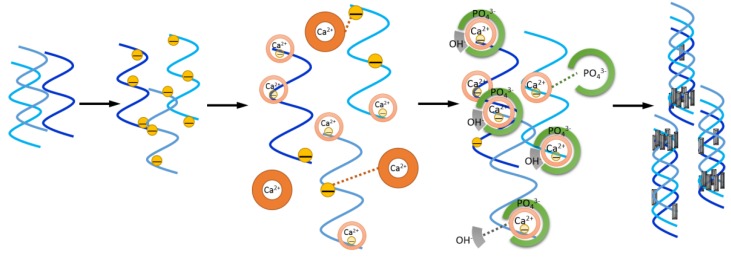
Schematic illustration of the biomimetic mineralization process of nHap/Col composite.

Figure 2 illustrated that the collagen fibrils re-assembled and the formation of Hap nanocrystallines occurred simultaneously to mimic *in vivo* physiological state under *in vitro* synthesis process. At first, the turbidity increase was caused by the fibrils assembly and simultaneous formation of amorphous phase of calcium phosphates. Electrostatic interaction could spontaneously occur between carboxyl groups of collagen with negative charges and calcium cations (Ca^2+^) in alkaline solution. Fibrillogenesis was facilitated by neutralizing the surface charge when the pH was close to the pI. This phenomenon slowed down the individual cluster precipitation of calcium phosphate crystal incoherent to organic phase. Subsequently, the amorphous calcium phosphate phase transformed into a crystalline phase, which resulted in a rapid increase of precipitate. Furthermore, mineralization was gradually reconstituted by phosphate addition through electrostatic attraction with Ca^2+^, which has strong binding affinity to collagen.

In the previous research studies, Hermann Ehrlich *et al.* mentioned that control over crystal growth by acidic matrix macromolecules is an important process in the formation of mineralized tissues. They modified the protein collagen by carboxymethylation using glucuronic acid and obtained more highly ordered structure [24]. The authors also developed dual diffusion membrane system for oriented crystal growth of octacalcium phosphate/hydroxyapatite on the biomimetically carboxymethylated collagen fibrils [25]. In a recent study conducted by Wolfgang Pompe *et al.*, they found that the directed growth of nanostructured octacalcium phosphate and the subsequent formation of hydroxyapatite (HAP) in collagen macrofibrils by a topotaxial transition for the biomimetic formation of biohybrid crystalline inorganic materials [26]. This suggests that biomolecular templates governed by metastable inorganic crystalline phases is a general feature of biomineralization processes.

**Figure 3:**
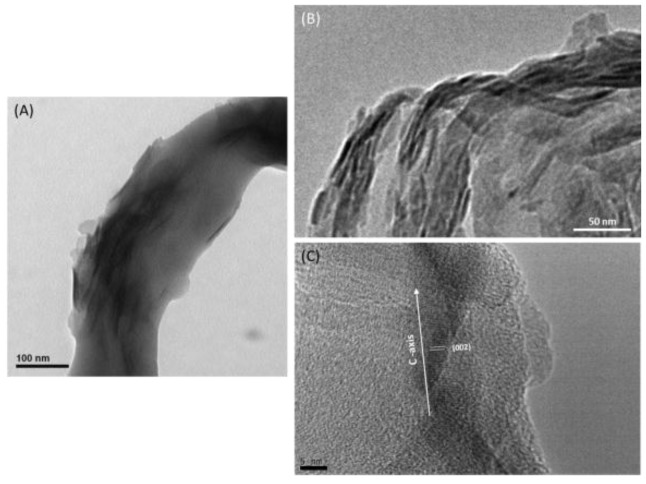
TEM images of mineralized nHap/Col composite material. (**A**) The c-axis of needle-like shape of Hap nanocrystals was specifically oriented along the longitudinal direction of the collagen fibrils; (**B**) each bundle consisting of Hap nanocrystals surrounded with Col fibrils; and (**C**) high-resolution TEM image with interplanar spacing corresponding to the (002) of Hap. Scale bar: (**A**) 100 nm; (**B**) 50 nm; and (**C**) 5 nm.

The newly formed mineral phase could be visualized and distinguished by transmission electron microscopy (TEM) after Hap mineralization (Figure 3). As a result, collagen self-assembly occurred simultaneously with the nucleation and growth of apatite crystals to achieve a bone-like ultrastructure. The nHap were closely associated with collagen fibers with the elongated fibril morphology. The epitaxial growth of nanoscaled Hap crystals on the biopolymer phase during collagen fibril reassembly were identified. From the TEM pictures (Figure 3B), each bundle consisting of many Col fibrils was surrounded with Hap nanocrystals. Collagen fibrils have been served as template directly for nucleation of Hap nanocrystals reorganization. The collagen framework could be partially mineralized by nHap to become a homogenous nanocomposite.

Moreover, Hap nanocrystals were embedded within collagen fibrils with their c-axis arranged roughly parallel to the long axis of the fibrils. The Hap crystals had needle-like shape with average dimensions of about 4 × 50 nm as identified on the collagen fibrils (Figure 3). According to Scherrer equation, the full width at half maximum (B) of the (002) diffraction peak is used to calculate the crystal size (d) of Hap. The crystallite size of synthesized Hap was 3.28 nm in agreement with HR-TEM image (Figure 3C). It exhibited parallel fringes with a spacing of 3.41 nm that was assigned to the (002) plane of Hap. This alignment of Hap nanocrystals was quite similar to the nanostructure observed on bone, indicating that the self-organization of Hap and Col could be reproduced *in vitro*.

### 2.2. Physicochemical and Biological Properties of nHap/Col Composite Scaffold

**Figure 4:**
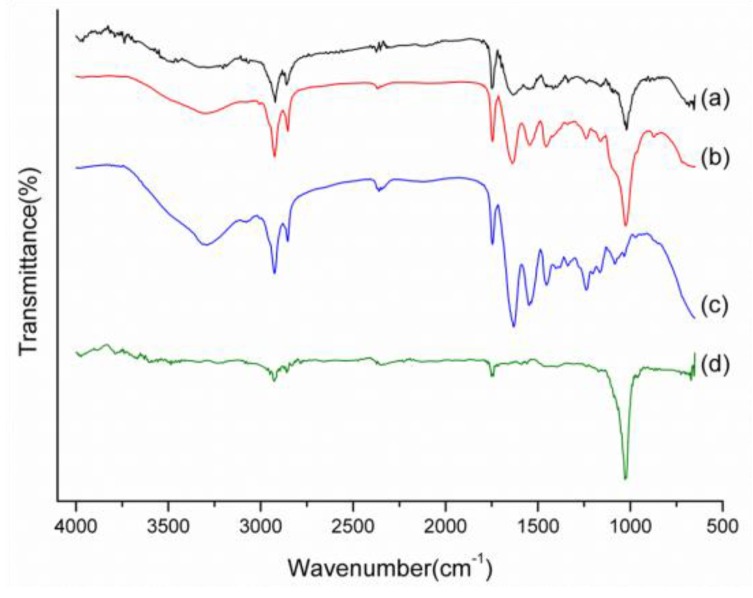
FTIR spectra of (**a**) nHap/Col scaffold after crosslinking; (**b**) nHap/Col scaffold without crosslinking; (**c**) pure collagen (Col); and (**d**) hydroxyapatite (Hap) materials.

The molecular structure of synthesized nHap/Col composite scaffold was examined by FTIR and those absorption bands could be identified as corresponding to collagen and hydroxyapatite, respectively (Figure 4). Featured stretching and bending modes of phosphate (PO_4_^3−^, P–O and O–P–O) for hydroxyapatite were exhibited in the region of 600–1100 cm^−1^. In addition, the distinctive peaks of collagen were observed, such as 1640 and 1740 cm^−1^ for the C=O group, 2850 and 2930 cm^−1^ for C-H stretching, 3230 cm^−1^ for N-H stretching, and 3200–3500 cm^−1^ for O–H intermolecular bonding. After crosslinking, aldehyde groups of GA could react with the amine groups of collagen to produce covalent imine bond (–C=N–) formation. The representative transmittance peak in crosslinked nHap/Col composite scaffold was obviously observed at the wavelength of 1690 cm^−1^. These findings indicated that the constituent of nHap/Col composite scaffold could still be maintained even after being covalently crosslinked by glutaraldehyde (GA).

**Figure 5:**
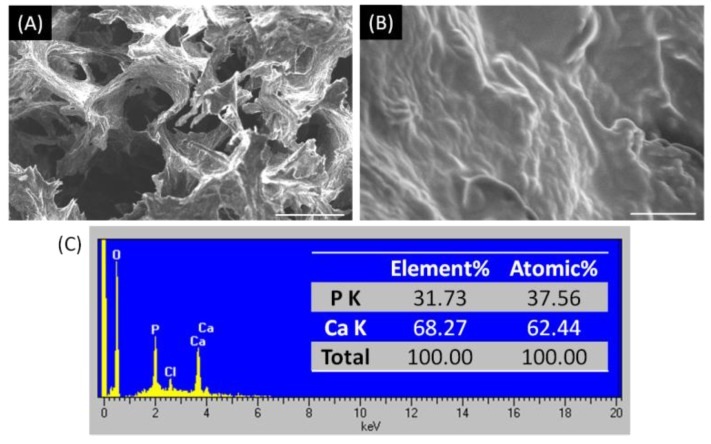
SEM images of nHap/Col composite scaffolds. Scale bar: (**A**) 100 μm; (**B**) 5 μm; (**C**) EDX spectrum of the selected area of nHap/Col nanocomposite scaffold; inset table presents the atomic ratio, percentage of the components in the nHap/Col composite.

The scaffold design has to develop a porous structure to achieve diffusion or permeability for mass transport and cell migration. The degree of interconnectivity is a critical factor as well as the pore size. The pore size is not exactly the cause of significant difference in bone growth [27]. It is generally accepted that a minimum pore size at least 100 μm is necessary for the porous implant materials to function well and necessary for osteoconduction [28]. The morphology of the nHap/Col composite scaffold was observed by SEM, shown in Figure 5. The nHap/Col composite scaffold showed open and interconnected porous structure with homogeneous distribution. The pore size and percentage of the nHap/Col composite in each preparation process (0.5 h, 3 h, 24 h) was 200 ± 30 μm (~85%). There was no significant difference in each group. The pore size was in the range of 200–300 μm, which met the requirements to serve as a bone scaffold. The pores are interconnected and could promote cell ingrowth and nutrient metabolism to support seeded cells.

As we know, Hap has excellent biocompatibility and bioactivity. It is osteoinductive as the mineral deposition and is slowly replaced by host bone after implantation [29,30]. Moreover, the major purpose of the Hap is to lead the deposition of the newly formed bone minerals to provide significantly higher construct mechanical stiffness at the end of cultivation [19]. The presence of Hap particulate deposits was evident shown in the higher magnification SEM images of the nHap/Col composite scaffold (Figure 5B). In order to confirm if the deposition of mineral was Hap contained in the composite scaffold, the elemental composition was traced by EDX analysis (Figure 5C). The spectrum showed the characteristic peaks of calcium and phosphate, suggesting the presence of both elements in the nHap/Col composite scaffold. The ratio of Ca/ P was about 1.66 on average. The value was closed to that of hydroxyapatite (Ca/P = 1.67). We also found that there was no dissociation mixture of Hap and collagen in the fabricated nHap/Col composite scaffold. This synthesized composite scaffold consisting of reconstituted collagen fibrils and nanoscaled Hap crystals possesses a similar structure to that of the nature bone tissue, which has been obtained through a self-assembling mineralization process.

**Figure 6:**
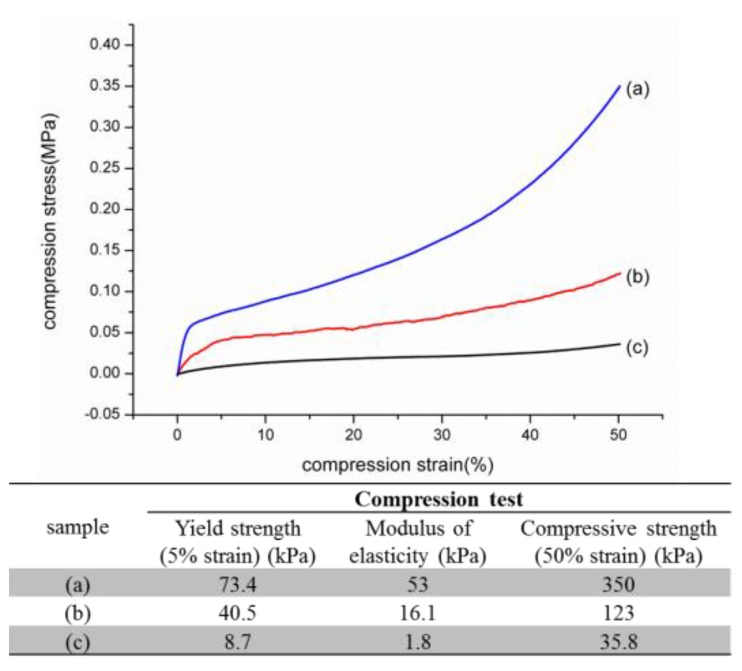
Stress-strain curves of nHap/Col composite scaffolds with different preparation process by compression test: (**a**) self-assembled mineralization method for 24 h reaction; (**b**) self-assembled mineralization method for 3 h reaction; (**c**) hydroxyapatite powder directly added into collagen slurry as simple mixture method. The mechanical properties of nHap/Col composite scaffold were summarized in the table.

Collagen and hydroxyapatite not only are among the most abundant class of biomineralized materials, but exhibit properties of both hard and soft matter; that is, combining the toughness of inorganic material and the flexibility of biological protein [31]. The representative stress-strain curves of the compression test for crosslinked nHap/Col composites scaffold were performed and shown in Figure 6. The strength properties (5% strain) and elastic modulus of biomimetic self-assembled nHap/Col composite scaffold could effectively improve ten-fold higher than that of the individual component mixing group. Moreover, the mechanical strength was enhanced with the increase of biomimetic precipitation reaction period. The nHap/Col composite scaffold used a biomimetic self-assembled approach to align Hap nanoparticles within collagen fibril, thus reinforcing the structure. The significant increase in the mechanical property of engineered bone constructs in the present study was likely due to the combined effect of the changes in the improved structure and the amount of minerals.

The mechanical properties depended on the effects of particle size, particle loading, particle/matrix interface adhesion, strength and toughness of composites [32]. Even though the compression endurance of an nHap/Col composite scaffold is considerably lower than those of native bone [33], it has been shown that potential improvement in mechanical properties can be achieved by increasing the incorporation amount of inorganic Hap in Col matrices, because strength depends on effective stress transfer between Hap precipitate (as additive) and Col fibril. The amount of Hap precipitate in the composite scaffold was increased along with the reaction time. Nevertheless, nonsymmetrical Hap precipitate often showed random orientated arrangement on collagen fibers during scaffold fabrication process. It was found that the nHap/ Col interface may cause stress raisers for crack growth and the appearance of separated phase if simply blending two components. The method of Hap deposition onto collagen in this study effectively reinforced composite scaffolds as suggested in the previous literature [13,29]. This result demonstrates the biomimetic self-assembled approach has the capability to tailor the mechanical properties of composite scaffolds before crosslinking.

**Figure 7:**
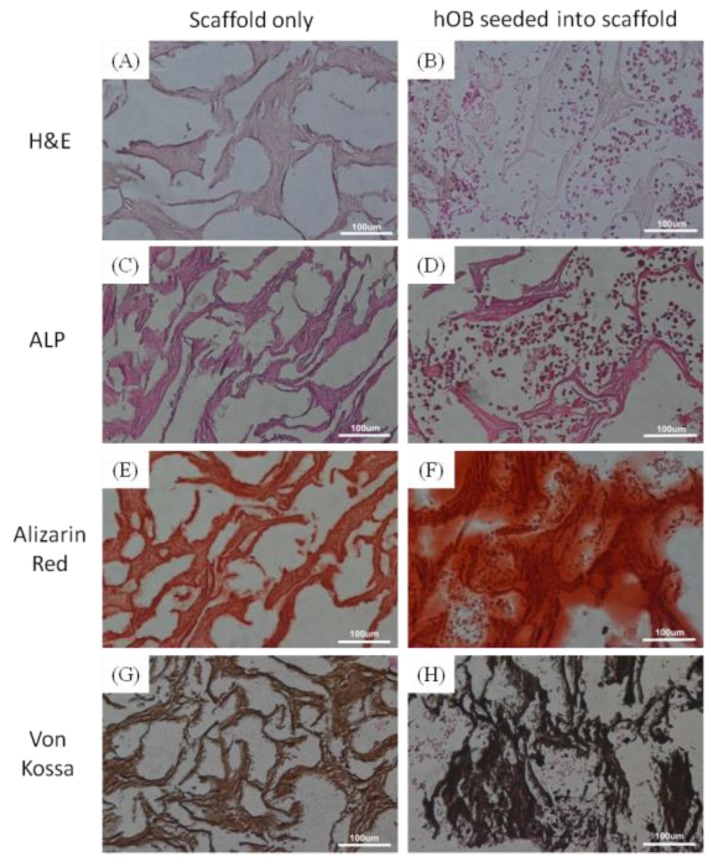
(**A**,**B**) H&E; (**C**,**D**) ALP; (**E**,**F**) Alizarin Red and (**G**,**H**) Von Kossa stained histological sections of nHap/Col composite scaffolds *in vitro*.

The nHap/Col composite scaffold possessed a homogenous porous structure which could be identified in Figure 7A. The main role of bone grafts is to provide a structural framework through which the host bone can infiltrate and regenerate new bone tissue for osteoconductive, osteoinductive or osteogenic characteristics [34]. The seeded hOB cells were well attached and survived in nHap/Col composite scaffolds in the result of H&E staining (Figure 7B). The histology pictures indicated that hOB cells can migrate into the nHap/Col porous scaffolds spreading with homogeneous distribution. To confirm the proliferation and differentiation of seeded hOB on nHap/Col composite scaffolds, ALP and Alizarin Red S were also examined after 3 weeks of cultivation. ALP, a cell surface protein, is known as an early marker for osteoblastic differentiation and new bone formation. Faint ALP stains on scaffold were detected in the culture group without cells, but the stains were much more extensive in the group cultured with hOB (Figure 7C,D). As seen in the ALP stain, the cell seeding group was strongly stained with Alizarin Red S as well (Figure 7F), while the scaffold without cells were not obvious detected (Figure 7E). Besides, von Kossa staining was also used to quantify mineralization in the reaction with phosphate groups on the nHap/Col composite scaffolds. Calcium deposits revealed strong performance on the nHap/Col scaffold after being cultured with hOB cells when compared to control groups (Figure 7G,H). A dense layer of mineralized matrix was observed throughout the nHap/Col composite scaffold. After 21 days of culture, the results demonstrated a dark von Kossa staining pattern within the nHap/Col composite scaffold, identifying the presence of calcium nodules within the sponge and therefore the mineralization of the extracellular matrix. On the other hand, mild brown color staining was observed in constructs without cells. This biochemical data confirmed the potential properties of nHap/Col composite scaffolds, which support cell attachment, growth, proliferation, and differentiation. After 3 weeks culture condition, the nHap/Col composite scaffolds not only could maintain their appearance serving as temporary substrate matrices but could also provide appropriate traits for improving the bone maturation and mineralization process. This has shown the promise of our developed nHap/Col nanocomposite as a synthetic bone graft substitute and tissue engineering scaffold.

## 3. Materials and Methods

### 3.1. Fabrication of Hap/Col Biomimetic Scaffold

The extraction of collagen was similar to and modified from that described in our previous work [35]. Briefly, porcine skin from a slaughter house was minced, defatted, and cleaned. The swollen skin was digested with pepsin (PB0688, BIO BASIC, Markham, ON, Canada) in HCl_(aq)_ (pH = 2) at room temperature for 24 h. The type I collagen (Col) was precipitated by adding NaCl_(aq)_ into the supernatant with 5% (w/v) final concentration followed by centrifugation. The extracted collagen was dialyzed and then obtained after freeze-drying. For hydroxyapatite (Hap) mineralization process on collagen fibrils, 1 g Col was dissolved in 30 mL 1 mM HCl_(aq)_ and then homogeneously dispersed in 0.109 M calcium hydroxide aqueous solution (Ca(OH)_2_, 1305-62-0, Riedel-de Haën, St. Louis, MI, USA). The equivalent volume of 0.0547 M phosphoric acid aqueous solution (H_3_PO_4_, 7664-38-2, J.T.Baker, Center Valley, PA, USA) was added to suspension at a rate of 3 mL/min for 0.5, 3 and 24 h incubation time at 37 °C under alkaline condition (pH = 9) adjusted by NaOH_(aq)_ throughout the synthesis process. The precipitation was suctioned under vacuum to collect the biomimetic nHap/Col composite and then freeze-dried to form 3D porous scaffold.

### 3.2. Characterization of Hap/Col Composite Scaffold

#### 3.2.1. X-ray Diffraction (XRD)

The crystal structure and lattice parameters of the Hap and Hap/Col composite scaffold were characteriszed and analyzed using XRD analysis (X’Pert PRO MPD, PANalytical, Almelo, Netherlands). The samples were scanned in the diffraction angle 2θ in the range of 10 to 75 with a scanning rate of 0.04°/min. The monochromatic x-radiation having a wavelength (λ) of 0.154 nm was used. The Scherrer equation was used to calculate the crystallite size. d = Kλ/B × cosθ, where d is the average diameter, K is the shape factor, B is the broadening of the full width at half maximum (FWHM) of the diffraction peak measured in radians, λ is the wavelength of the x-rays and θ is the Bragg’s diffraction angle. The FWHM of the (002) diffraction peak is used to calculate the crystal size of the nanoparticle in the later experiment. All samples were prepared as fine powders before the analysis. The phase identification was performed with a reference to the JCPDS No. 09-0432 data card.

#### 3.2.2. Fourier Transform Infrared Spectroscopy (FTIR)

The chemical bonds and functional groups of Hap/Col composite with or without glutaraldehyde (GA, A17876, Alfa Aesar, Ward Hill, MA, USA) crosslinking were identified and characterized by FTIR spectra (JASCO 410, Tokyo, Japan). For each spectrum, 32 scans between the wavelengths of 650 to 4000 cm^−1^ were recorded in the transmission mode by the potassium bromide method.

#### 3.2.3. Thermal analysis

The changes in physical and chemical properties were measured as a function of increasing temperature (with constant heating rate) by differential scanning calorimetry (DSC) coupled with thermogravimetric analysis (TGA, SDT 2960, TA Instruments, New Castle, PA, USA). Samples were conducted with a scanning temperature from room temperature up to 1000°C at a heating rate of 10°C/min in air flow using empty platinum pan as a reference.

#### 3.2.4. Scanning Electron Microscopy (SEM) and High-Resolution Transmission Electron Microscopy (HR-TEM)

The structural morphology and chemical composition of the scaffolds were examined by SEM (S-3000N, Hitachi, Tokyo, Japan) incorporated with energy-dispersive X-ray spectroscopy (EDX, JSM-5600, JEOL, Tokyo, Japan) analysis. Specimens were only sputtered with thin gold coating before observation. The orientation of Hap precipitates allied with collagen fibril during mineralization was investigated using HR-TEM (JEM-2100, JEOL, Tokyo, Japan) for more details.

#### 3.2.5. Mechanical Test

Compressive properties of the porous scaffolds was measured at room temperature using a universal testing machine (8848 microtester, Instron, Buckinghamshire, UK) with 50 N of load cell capacity. The dry cylindrical porous scaffolds at a diameter of 12 mm and a height of 25 mm were used as the testing specimens and compressed at a rate of 0.6 mm/min. The test ended after the scaffold sustained 50% strain. The modulus of elasticity (E) was calculated as the slope of stress *vs.* strain curve at 5% strain of the linear region. All results were the average of three measurements.

### 3.3. Histological Analysis

Human osteoblasts (hOB) were obtained from the upper femur of the patients undergoing total hip replacement surgery for osteoarthritis from National Taiwan University Hospital in compliance with hospital regulations. The residual connective tissue and blood within harvested bone were rinsed by sterile PBS irrigation several times. The hOB were then harvested with 0.2% type I collagenase for overnight incubation and then cultured in Minimum Essential Medium alpha (11900024, Gibco, Carlsbad, CA, USA) supplemented with 10% fetal bovine serum (04-001-1, Biological Industries, Cromwell, CT, USA), 100 U/mL penicillin and 100 µg/mL streptomycin (15140122, Gibco, Carlsbad, CA, USA) in a humidified incubator at 37 °C, 5% CO_2_. Passage numbers of hOB used in the experiments were between 2 and 4. One million hOB cells were seeded into Hap/Col composite disc scaffold (5 mm in diameter, 2 mm in height) for 2 h to allow for cell attachment, and then cultured in growth medium for 3 weeks. After that, bone substituent was washed, fixed, dehydrated, and sectioned. The sliced (~5 μm) specimens were examined and stained by hematoxylin and eosin (H&E) to reveal the distribution of cells throughout the composite matrix. Alkaline phosphatase (ALP), Alizarin Red S and von Kossa were used to evaluate osteoblast proliferation during *in vitro* bone formation, cell-mediated calcium deposition and quantify mineralization within scaffolds, respectively.

### 3.4. Statistically Analysis

All data are expressed as the mean ± standard error and was analyzed by one-way analysis of variance (ANOVA). Statistical significance was accepted at a level of *p* < 0.05.

## 4. Conclusions

In the present study, we have investigated the potential for self-assembled mineralization of nHap/Col composite scaffolds as well as the improvement of mechanical properties and osteogenesis to enhance the *in vitro* formation of bone-like tissues. A biomimetic three-dimensional hybrid nHap/Col scaffold with aligned hydroxyapatite nanocrystals associated with collagen fibrils using a self-assembling approach for bone reconstruction has been developed. The nanostructure of the nHap/Col composite scaffold, which consists of a c-axis of Hap nanocrystals aligned along Col fibers has similar distinctive features to those of natural bone. The nHap/Col scaffold also possesses good bioactivity for hOB proliferation and secretion of bone-specific proteins. In summary, we successfully demonstrate that oriented nHap/Col composite scaffold has great potential applications in bone tissue engineering.
